# Supplementary material for: A Critical View of the Application of the APEX Software (Aqueous Photochemistry of Environmentally-Occurring Xenobiotics) to Predict Photoreaction Kinetics in Surface Freshwaters
Source: Molecules. 2019 Dec 18;25(1):9. doi: 10.3390/molecules25010009 (PMC7017383; doi:10.3390/molecules25010009)
Supplement: Supplementary file 1 [file molecules-25-00009-s001.zip › 3.2.4_gcc-4.4.0.zip!/3.2.4_gcc-4.4.0/3.2.4_gcc-4.4.0/doc/octave/html/interpreter/General-Guidelines.html]

General Guidelines - Untitled


Next: Octave Sources (m-files),
Previous: How to Contribute,
Up: Contributing Guidelines


---

### D.2 General Guidelines

All Octave's sources are distributed under the General Public License (GPL).
Currently, Octave uses GPL version 3. For details about this license, see
http://www.gnu.org/licenses/gpl.html. Therefore, whenever you create a
new source file, it should have the following comment header (use appropriate
year, name and comment marks):

```
     ## Copyright (C) 1996, 1997, 2007 John W. Eaton <jwe@octave.org>
     ##
     ## This file is part of Octave.
     ##
     ## Octave is free software; you can redistribute it and/or
     ## modify it under the terms of the GNU General Public
     ## License as published by the Free Software Foundation;
     ## either version 3 of the License, or (at your option) any
     ## later version.
     ##
     ## Octave is distributed in the hope that it will be useful,
     ## but WITHOUT ANY WARRANTY; without even the implied
     ## warranty of MERCHANTABILITY or FITNESS FOR A PARTICULAR
     ## PURPOSE.  See the GNU General Public License for more
     ## details.
     ##
     ## You should have received a copy of the GNU General Public
     ## License along with Octave; see the file COPYING.  If not,
     ## see <http://www.gnu.org/licenses/>.
```

Always include ChangeLog entries in changesets. After making your source
changes, record and briefly describe the changes in the nearest ChangeLog file
upwards in the directory tree. Use the previous entries as a template. Your
entry should contain your name and email, and the path to the modified source
file relative to the parent directory of the ChangeLog file. If there are more
functions in the file, you should also include the name of the modified function
(in parentheses after file path). Example:

```
     2008-04-02  David Bateman  <dbateman@free.fr>
     
             * graphics.cc (void gnuplot_backend::close_figure (const
             octave_value&) const): Allow for an input and output stream.
```

The ChangeLog entries should describe what is changed, not why. Any
explanation of why a change is needed should appear as comments in the
code, particularly if there is something that might not be obvious to
someone reading it later.

The preferred comment mark for places that may need further attention is FIXME.
